# Drug-Rich Phases Induced by Amorphous Solid Dispersion: Arbitrary or Intentional Goal in Oral Drug Delivery?

**DOI:** 10.3390/pharmaceutics13060889

**Published:** 2021-06-15

**Authors:** Kaijie Qian, Lorenzo Stella, David S. Jones, Gavin P. Andrews, Huachuan Du, Yiwei Tian

**Affiliations:** 1 Pharmaceutical Engineering Group, School of Pharmacy, Queen’s University Belfast, 97 Lisburn Road, Belfast BT9 7BL, UK; kqian02@qub.ac.uk (K.Q.); D.Jones@qub.ac.uk (D.S.J.); g.andrews@qub.ac.uk (G.P.A.); 2 Atomistic Simulation Centre, School of Mathematics and Physics, Queen’s University Belfast, 7–9 College Park E, Belfast BT7 1PS, UK; l.stella@qub.ac.uk; 3 David Keir Building, School of Chemistry and Chemical Engineering, Queen’s University Belfast, Stranmillis Road, Belfast BT9 5AG, UK; 4 School of Pharmacy, China Medical University, No.77 Puhe Road, Shenyang North New Area, Shenyang 110122, China; 5 Laboratory of Applied Mechanobiology, Department of Health Sciences and Technology, ETH Zurich, Vladimir-Prelog-Weg 4, 8093 Zurich, Switzerland; 6 Simpson Querrey Institute, Northwestern University, 303 East Superior Street, 11th floor, Chicago, IL 60611, USA

**Keywords:** amorphous solid dispersion, drug-rich phase, liquid-liquid phase separation, permeability enhancement, bioavailability enhancement

## Abstract

Among many methods to mitigate the solubility limitations of drug compounds, amorphous solid dispersion (ASD) is considered to be one of the most promising strategies to enhance the dissolution and bioavailability of poorly water-soluble drugs. The enhancement of ASD in the oral absorption of drugs has been mainly attributed to the high apparent drug solubility during the dissolution. In the last decade, with the implementations of new knowledge and advanced analytical techniques, a drug-rich transient metastable phase was frequently highlighted within the supersaturation stage of the ASD dissolution. The extended drug absorption and bioavailability enhancement may be attributed to the metastability of such drug-rich phases. In this paper, we have reviewed (i) the possible theory behind the formation and stabilization of such metastable drug-rich phases, with a focus on non-classical nucleation; (ii) the additional benefits of the ASD-induced drug-rich phases for bioavailability enhancements. It is envisaged that a greater understanding of the non-classical nucleation theory and its application on the ASD design might accelerate the drug product development process in the future.

## 1. Introduction

Modern drug discovery has excelled through powerful computational chemistry and high-throughput screening technologies. However, it is widely accepted that efficacious delivery of these new chemical entities (NCEs) can be extremely difficult [1,2,3]. The oral absorption of a drug is a complex process that can be affected by various factors such as physicochemical factors of the drug and formulation, and physiological factors of the patients [4]. From the formulation and drug delivery perspectives, the Biopharmaceutics Classification System (BCS) is established based on two key parameters, the drug solubility in the media in relation to its maximum dose and the drug permeability through the gastrointestinal membrane. Statistically, approximately 40% of the marketed drugs and up to 90% of the new drug candidates have been revealed to be poorly soluble in aqueous media (BCS II and IV) [5,6,7]. More importantly, around 25% to 40% of all approved drugs are also suffering from low permeability (BCS IV) [6,7,8]. To combat these challenges, various formulation strategies such as amorphous solids [9,10], nanocrystals [11,12], liposomes [13,14], micro/nano-emulsifying systems [15] and co-crystals [16] have been developed.

Amorphous solids and amorphous solid dispersions (ASDs) have drawn increased attention due to their continuous commercial successes in the past decade [17]. The high free energy state and the disordered structure of the amorphous solids can lead to a remarkable enhancement of the drug solubility as well as the dissolution rate. For example, amorphous glibenclamide displayed 14 times higher solubility than its crystal form in an aqueous buffer [18]. The apparent solubility of amorphous indomethacin is higher than the γ-crystal API over the temperature range from 5 to 45 °C [18]. Amorphous pranlukast enhanced apparent solubility approximately 5.8 times in water and 19.4 times in phosphate buffered saline, compared with the crystal substance [19]. However, the inherent instability of amorphous solids remains the major concern in terms of its wider adoption in modern medicine.

Amorphous solid dispersions (ASDs) are, in general, homogeneous dispersions of amorphous drug molecules within a solid excipient [20]. To overcome the instability of amorphous drugs caused by the high free energy, certain polymers are utilized as the anti-plasticizer and/or the stabilizer to maintain the amorphous structure of the drug molecules during storage [21,22,23]. Polymers can increase formulation stability through various mechanisms such as the physical barrier, configurational entropy, molecular mobility, chemical potential, glass transition temperature and drug-polymer interaction [24]. Nontoxic polymers including polyvinylpyrrolidone (PVP), hypromellose (HPMC), polyvinylpyrrolidone/vinyl acetate (PVP/VA), hypromellose acetate succinate (HPMCAS) have been approved to use as excipients by the United States Food and Drug Administration (FDA) for oral dosage forms [20,24]. The ASD products approved by FDA in the last 5 years are briefly summarized in Table 1, indicating an ascending phase of this technology for wide adoptions in the pharmaceutical industry [20,24,25].

It should also be highlighted here that the choices of excipients for the ASD system are not narrowly limited to the polymers. Indeed, certain small molecular additives, drugs [30,31,32,33] or ionic liquids (ILs) [34,35] have also been explored as the excipients in ASD design [36]. For example, the co-amorphous drug system of atorvastatin calcium-carvedilol and atorvastatin calcium-glibenclamide exhibited greater solubility than that of single amorphous component [33]. Amino acids, such as arginine and phenylalanine, were also implemented to stabilize indomethacin and enhance its solubility, reaching a level approximately 200 times greater than its pure amorphous counterpart [37]. Ionic liquid, defined as salts with melting/glass transition temperatures below 100 °C, can also be used to solubilize the drug [38,39]. The targeted biological properties of the drug-IL system have been categorized as the third evolution of ILs in the history of their development [40]. In the context of ILs’ pharmaceutical application, the ideal drug-ILs are expected to be liquid at the body temperature for improved dissolution properties [41,42]. Solubilized drug-ILs were revealed to increase the drug’s apparent solubility, dissolution rate and membrane transport properties for oral administration [34,35,43,44,45]. Several excellent reviews have already been published for the solubilized drug-IL amorphous systems, demonstrating the promising future of this molecular complex approach in the pharmaceutical field [41,42,46].

Size reduction is another important technique to increase the dissolution and oral absorption of poorly water-soluble drugs. The synergetic effects of size reduction and amorphous structure are expected to further improve the performance of the formulation. A range of existing scalable manufacturing methods can be used to produce amorphous nano-sized drugs, such as microfluidics, ultrasonication, antisolvent precipitation, electro-spraying, and the supercritical fluid process [47,48,49,50,51]. Apart from amorphous drug nanoparticles prepared during formulation processes, various drug-rich amorphous nanoparticles or nanodrops were also reported during the dissolution of the ASDs. High apparent drug solubility and bioavailability enhancement were commonly associated with the presence of such drug-rich phases [52,53,54,55,56,57]. However, the formation of these phases and their corresponding stability in the solution are still poorly understood. Hence, we suggest that a better understanding of such drug-rich phases will be highly beneficial to predict the performance of different formulation designs and thereby rationalize them for the final implementation of ASD formulation strategy in industry.

In this review, we highlight: (i) the thermodynamics and kinetics associated with the formation of drug-rich phases from the dissolution (Section 2); (ii) in vitro permeability enhancement and in vivo bioavailability enhancement in the context of ASD formulation (Section 3). Other aspects associated with the drug-rich phases, such as the dissolution model [58], the liquid-liquid phase separation (LLPS) identification and screening technique [59], and the impact of surfactants and polymers in the nanodroplet uptake [60,61], can be found in other excellent studies in the literature.

## 2. Dissolution Pathways of ASD in Water

ASD has been widely revealed as a promising strategy in improving drug bioavailability and therapeutic windows for poorly water-soluble drugs. However, a mechanistic understanding of its phase separation process during dissolution and storage is still difficult to obtain. In this section, we elucidate the solubility, dissolution and several possible phase separation pathways of pure drug and drug-excipient ASD in water from a simple thermodynamic viewpoint. We would like to imply that the non-classical nucleation pathways discovered with recent advances in biomineralization and protein precipitation, including the formation of different metastable transient phases and their subsequent transformation into crystals, might be a suitable model to describe the phase separation process during excipient-assisted amorphous drug dissolution [62,63,64].

### 2.1. Thermodynamics of the Pure Drug in the Dissolution

**Figure 1:**
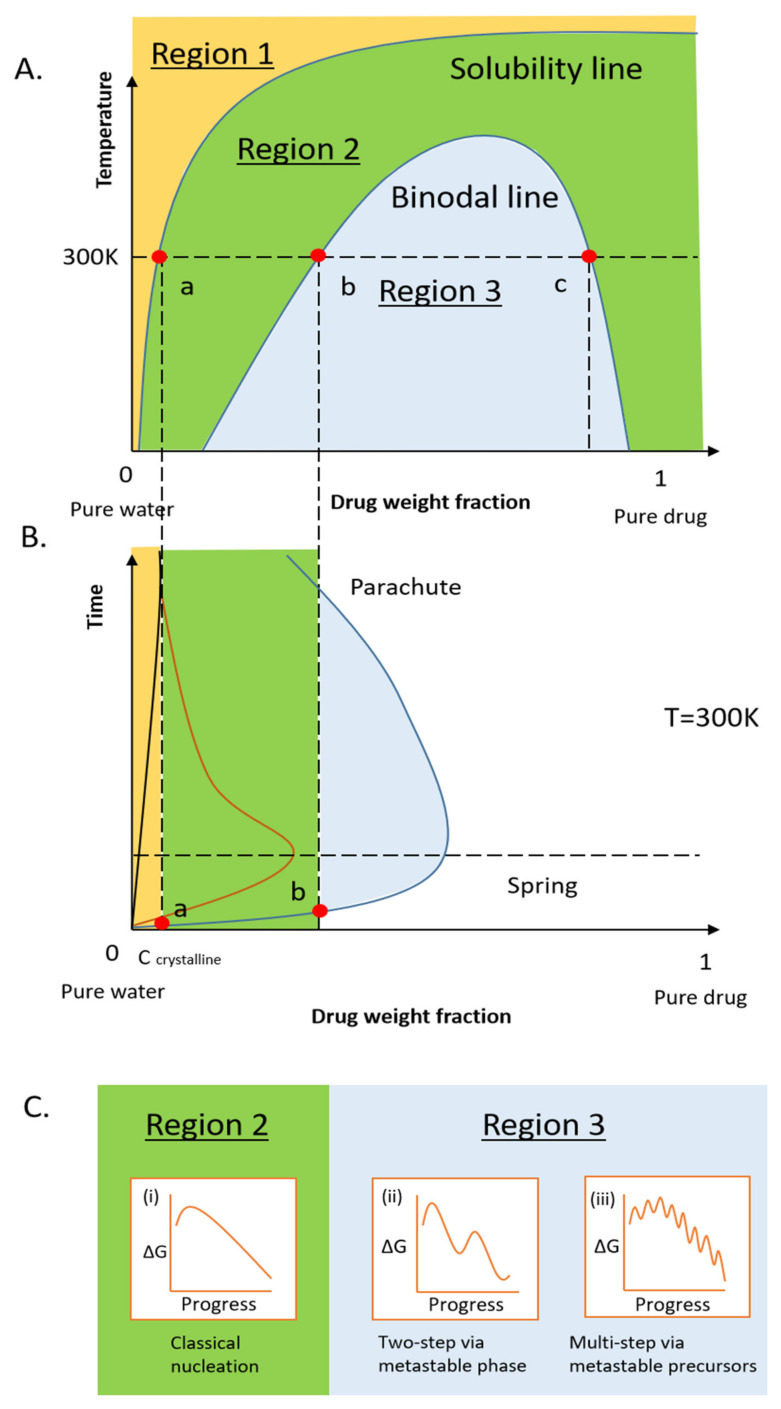
(**A**) Schematic temperature-composition phase diagram of the drug-water binary system, (**B**) the schematic dissolution diagram of a crystalline drug (the black solid curve), pure amorphous drug “spring” (the brown solid curve) and ASD formulation “spring and parachute” (the blue solid curve). (**C**) Gibbs free energy landscapes of dissolved drug molecules forming a stable bulk crystal through (i) classical nucleation and growth pathway, (ii) the two-step pathway via the metastable drug-rich liquid phase, and (iii) the aggregation of thermodynamically metastable particles and possible pathways [64]. Point a is the intersection of the solubility line and the horizontal temperature line, which reflects drug crystalline solubility at this temperature. Points b and c are intersections of the binodal line, indicating the drug and water weight fraction in drug-rich phases and drug-lean phases.

A typical drug-water binary phase diagram is depicted in Figure 1A. The solubility of a crystalline drug in an aqueous solution is determined by the thermodynamic equilibrium between the dissolved free drug molecules and the non-dissolved crystals in the solution. The equilibrium boundary can be represented by the crystalline solubility line in the phase diagram [65]. The drug crystalline solubility (C_crystalline_) at 300 K could be obtained using the intersection of the solubility line at this temperature (Figure 1, point a). If the number of free drug molecules dissolved in water is below the corresponding value of C_crystalline_ (Figure 1A, region 1), the solution is homogeneous, and no drug molecules will precipitate out from the solution. By contrast, if the amount of dissolved drug is above C_crystalline_ (Figure 1A, region 2 and region 3)—specifically, if it is supersaturated with respect to the solubility of the crystalline drug—the solution tends to phase separate due to its thermodynamic instability and, therefore, the drug precipitates out. When the supersaturation is moderate (region 2), the system undertakes a classical nucleation pathway to reduce its free energy, where solid crystalline nuclei exceeding the critical size form through the thermal fluctuation of the solution and subsequently grow via the molecule-by-molecule attachment process (Pathway (i) in Figure 1C). As a result, the free drug concentration in the solution will eventually decrease to the value of C_crystalline_.

Remarkably, if the supersaturation of drug solution is very high, drug-rich transient liquid or amorphous solid phases are often observed to form in the solution prior to the formation of crystalline drugs [66]. These drug-rich phases were suggested to form through the liquid-liquid phase separation (LLPS) of the drug-water binary system only if the concentration of free drug molecules in solution exceeds the critical value at the binodal line. Indeed, similar solute-rich liquid phases generated by the LLPS process have been extensively reported in the protein, inorganic ion and organic molecule solutions [67]. As the free energy barrier to form metastable liquid phases is smaller than that to form crystalline solid nuclei, a two-step nucleation pathway, via the formation of metastable drug-rich liquid phases (pathway (ii) in Figure 1C), is thermodynamically favored over the classical one. More recently, a wide variety of metastable transient precursors including complexes, prenucleation clusters, liquid phases, amorphous solid particles and nanocrystals have been demonstrated in the inorganic system [64,68]. The presence of one or multiple metastable precursors in the crystallization process is expected to further alter its free energy landscape and, therefore, the crystallization pathways. For example, when the supersaturation of solution is sufficiently high, the formation of metastable solute clusters or complexes and their subsequent aggregation could become thermodynamically favored, thereby rendering the free energy landscape with multiple local minima (pathways (iii) in Figure 1C) [64]. Interestingly, a recent study demonstrates that the liquid phases are likely the dynamic aggregates of clusters in the CaCO_3_ system, suggesting a possible correlation between different non-classical nucleation pathways [69]. However, more investigations must be conducted to identify the possible transient precursor species generated during the dissolution process of ASD and their corresponding nucleation pathways.

Using the above phase diagram, the general behaviors of the dissolution of the drug in water as well as the subsequent phase separation of the drug-water system can be qualitatively estimated. For example, if crystalline drug particles with an amount above the value of corresponding C_crystalline_ are suspended into the water, the concentration of free drug in aqueous solution gradually increases and eventually reaches the crystalline equilibrium solubility value (Figure 1, point a) with an extended time, as illustrated in a black solid curve in Figure 1B. If amorphous drug particles are suspended in solution, the drug concentration can temporarily reach beyond the value of C_crystalline_ because of their higher Gibbs free energy and, therefore, can achieve higher solubility relative to their crystalline counterparts [70]. The resulting high supersaturation of drug solution will lead to phase separation of the system. This phase separation process is typically expected to undergo via a classical nucleation and growth process (Figure 1, region 2), in which the drug concentration eventually approaches to the value of C_crystalline_. Hence, the pure amorphous drug is often revealed to display a “spring” dissolution profile (Figure 1B, brown curve). Interestingly, some recent studies suggested that the critical concentration of drug at the binodal line (Figure 1A, point b) is similar to the intrinsic solubility of amorphous drugs [71]. In line with this suggestion, if the dissolution of pure amorphous drug particles reaches the thermodynamic equilibrium state in the solution, we should expect LLPS to occur in the system. However, as the supersaturated drug solution readily forms crystalline nuclei due to its metastability and the dissolution rate of amorphous drug particles is not sufficiently high, this critical concentration might be difficult to achieve in a pure amorphous drug-water system. Indeed, the experimental observations of LLPS are typically reported in the systems where they rapidly enter the binodal regime with minimum interference of the nucleation-growth pathway using alternative strategies. For example, C_crystalline_ in the solution is rapidly decreased by switching the pH [72], temperature [73] and solvent [66] of a solution, resulting in an abrupt elevation of supersaturation extent of the system. Alternatively, certain polymeric or small molecular additives can be added into the system to delay the nucleation-growth pathway during the dissolution of the amorphous drug. In addition, as demonstrated in the biomineralization field, the amorphous phases of even the same mineral could possibly possess a broad range of local structures and water contents, which in return reflects on their solubility, dissolution and kinetic stability [68,74]. By analogy, the presence of possible structural or compositional variations in the drug-rich metastable phases might also influence the dissolution, solubility and therefore the phase separation phenomenon of the amorphous drug in water, yet the experimental evidence is still missing.

### 2.2. Thermodynamics of ASD in Dissolution

**Figure 2:**
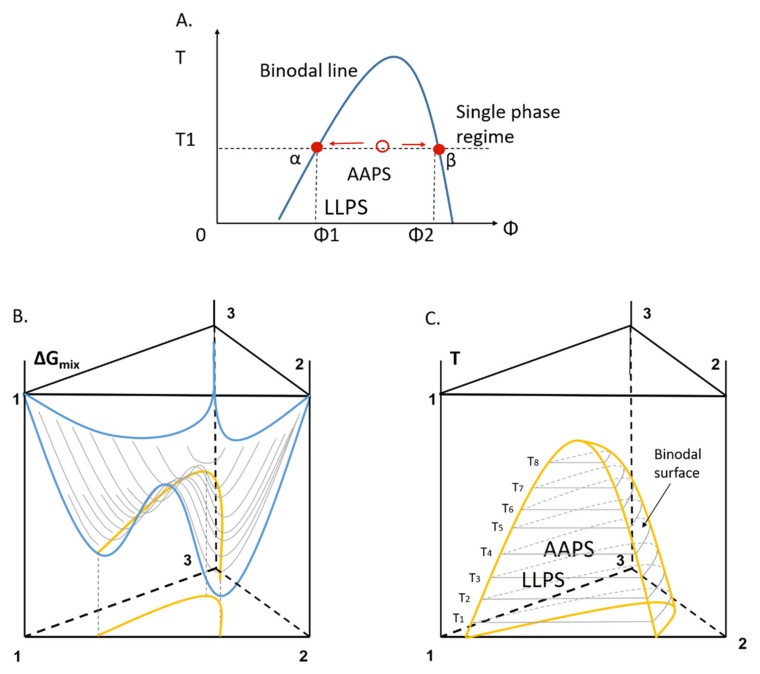
(**A**) Schematic diagram of the binodal line reflecting the boundary of a single homogeneous phase and AAPS/LLPS for an amorphous-amorphous binary system consisting of an upper critical solution temperature (UCST). (**B**) Gibbs free energy landscape of drug-polymer-water ternary system at a constant temperature, where the solid blue line describes the free energy surface, and the solid yellow line represents the binodal line at this temperature. (**C**) Composition-temperature phase diagram of the ternary system. The binodal surfaces are labeled with the solid yellow lines (components 1, 2 and 3 represent drug, polymer, and water, respectively).

In the ASD system, different amorphous polymeric or small molecular excipients are utilized to stabilize the amorphous drug during the storage as well as to enhance the solubility and dissolution rate of the drug in solution [21,22,23,24]. While amorphous drug molecules are considered to be homogeneously dispersed within the excipients in an ideal ASD system, an amorphous-amorphous phase separation (AAPS) phenomenon is often observed during the manufacturing or storage due to the imperfect miscibility of drug and excipients [29,75,76,77]. The AAPS phenomenon can be normally interpreted by the schematic phase diagram of ASD illustrated in Figure 2A, where the solid blue line indicates the binodal line. At this binodal line, the single homogeneous ASD phase splits into drug-rich and drug-lean amorphous phases. Theoretically, the drug volume fractions of these two phases are expected to be Φ1 and Φ2 at the specific temperature T1 under the equilibrium state (Figure 2A). However, in most ASD systems, particularly those with polymers as the excipients, the restricted mobility of polymer chains hinders the system to reach the equilibrium state. Indeed, the AAPS phenomenon is more pronounced at lower amounts of excipient or at a temperature range above the glass transition temperature of the system, where the mobility of drug and excipient molecules is higher [75].

Recently, more investigations have been carried out to correlate AAPS to the stability of ASD systems during their preparation and storage. In particular, the impacts of water/solvent on the AAPS process have been extensively studied in cases in which ASD is stored in a humid condition [76,78,79,80,81,82,83]. The Gibbs free energy surface of the drug-excipient-water ternary system at a constant temperature and the corresponding binodal lines at various temperatures in the phase diagram may be schematically illustrated in Figure 2B,C. The composition of ASD, temperature and water amount are expected to affect the Gibbs free energy landscape of the ternary system, thereby influencing the AAPS process and the stability of ASD. More recently, a more quantitative thermodynamic model was reported for the drug-polymer-water ternary system based on the Flory-Huggins (F-H) theory [65]. This ternary phase model described the drug-polymer-water interaction and the phase separation situation at various temperatures and composition concentrations. Similarly, important tool such as Perturbed-Chain Statistical Associating Fluid Theory (PC-SAFT) modelling has also been used for the quantitative analysis of these systems [84]. Other hybrid models for drug-polymer binary systems and the subsequent extension of ternary or quaternary systems have been reported for the design and understanding of ASD formulations [85,86,87,88]. The combination of these insightful thermodynamic and kinetic models will certainly provide a more informative guide for the AAPS process in presence of moisture/solvent and therefore a better understanding of the ASD stability during its preparation and storage.

With the same concept, a similar drug-excipient-water ternary system can be obtained if ASD is dissolved into water, where the amount of water in this system is nevertheless significantly higher than that absorbed from the moisture. Interestingly, the formation of metastable drug-rich amorphous phases, in the form of either nanoparticles or nano-sized liquid phases, are more frequently observed in this system compared to the pure drug-water binary system, as summarized in Table 2. These results indicate that the presence of excipients can play an important role in the phase separation of the drug in solution. For example, one can postulate that the introduction of the AAPS process of drug-excipient system to the LLPS process of drug-water system would result in a non-inferior [89] or lower supersaturation extent [88]. However, many hydrophilic polymer excipients can significantly enhance the rates of hydration and intrinsic dissolution performances of the drug from ASD [90]. It is evidenced that these approaches can facilitate the system to cross the binodal boundary and form different metastable drug-rich amorphous precursors following the non-classical nucleation pathways. Indeed, it remains unknown how the polymeric excipients assist the LLPS of the drug after ASD is exposed to an excess amount of water. Additionally, certain excipients can effectively prevent the aggregation of these metastable phases or reduce the nucleation and growth rate of drug crystals within the drug-rich metastable phases [23,91]. As a result, the kinetic stability (parachute stage), namely the extended time associated with the high apparent drug concentration in the solution, of the drug-rich metastable phase could be remarkably increased. This kinetic stabilization effect enables researchers to access the metastable drug-rich phases using the current characterization tools and therefore more frequently observe their presence. Moreover, the thermodynamically favored formation of drug-rich metastable phases and the kinetic stabilization of these phases during the dissolution process of ASD can eventually lead to a “spring and parachute” concentration profile in dissolution assays (Figure 1B, the blue solid curve). In this case, the drug remains at a much higher concentration for a more extended time in solution, as compared to that observed in the dissolution of pure crystalline or amorphous drug. Thus, such forced steps have been frequently used to screen the potential pharmaceutical excipients for such purposes.

### 2.3. Kinetic Stability of Drug-Rich Phases

If the drug-rich metastable phases are kinetically stabilized by certain excipients for a considerable amount of time, the high concentration of a drug in a solution can significantly promote the drug’s oral absorption. Consequently, the “reservoir effect” of drug-rich phases followed by the drug replenishing may release drug molecules to the medium without the precipitation of crystals. These application potentials of drug-rich phases have driven extensive research on the kinetics of their transformation and the corresponding kinetic stability against the transformation. Different characterization tools, such as ultraviolet-visible (UV) spectroscopy [92,93], fluorescence spectroscopy [71,94], dynamic light scattering (DLS) [72,95], atomic force microscopy (AFM) [96], scanning electron microscope (SEM) [95], transmission electron microscope (TEM) [96,97], nuclear magnetic resonance (NMR) [98], tunable resistive pulse sensing (TRPS), analytical ultracentrifugation (AUC) and liquid cell TEM have been utilized to access the information at different stages of drug-rich phases [99,100,101,102,103].

**Figure 3:**
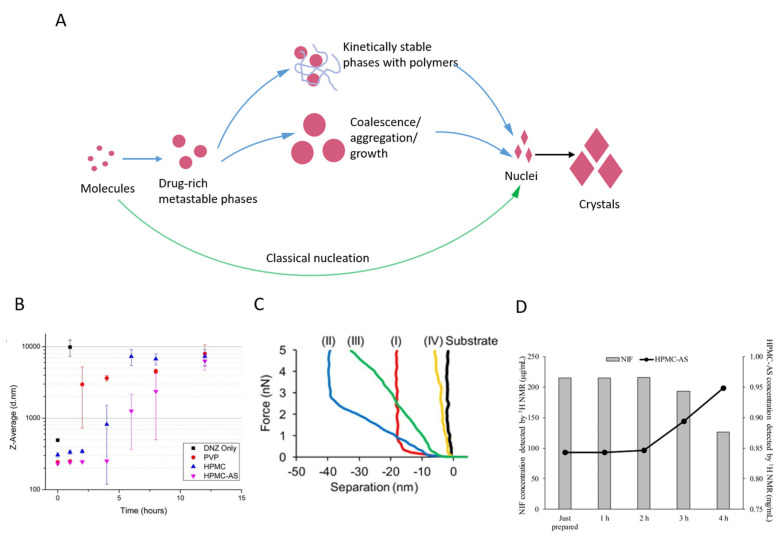
(**A**) Schematic illustration of the possible nucleation pathways (classical or non-classical) of drug in a solution. (**B**) Danazol Z—average diameters over time for systems with or without polymers. Reproduced with permission from [23], American Chemical Society, 2014. (**C**) Four schematic AFM force-distance curves for probucol (PBC)-HPMC-dodecyl sulfate systems over the storage time, (I) 1 d, (II) 2 d, (III) 4d, and (IV) 7d. Reproduced with permission from [106], American Chemical Society, 2015. (**D**) Solution 1H NMR detectable nifedipine and HPMCAS concentration over time. Reproduced with permission from [107], American Chemical Society, 2017.

As the transient drug-rich phases are thermodynamically metastable, they would eventually transform into thermodynamically stable crystals. Several possible transformation pathways that have been revealed are schematically summarized in Figure 3A, even though a full picture of drug-rich phase transformation is still missing. The drug-rich phases might continue to grow, coalesce, or aggregate to further reduce the free energy of the system [66,104]. For example, the diameter of drug-rich phases in the supersaturated danazol aqueous solution was revealed to increase over time when monitored using dynamic light scattering (DLS) techniques (Figure 3B) [23]. Similarly, Ralm et al. showed that the metastable amorphous particles formed in the supersaturated phenytoin-HPMCAS aqueous solution aggregated into irregularly shaped nanoparticles prior to the formation of crystals, as measured with Cryo-TEM and SAXS [105]. Although the growth, coalescence or aggregation of metastable phases is favored over the formation of crystalline nuclei due to the smaller free energy barrier, the nucleation events will still occur, most likely within the drug-rich phases where a higher supersaturation with respect to the crystalline solubility is present. These nucleation events will trigger the transformation of drug-rich metastable phases into thermodynamically stable crystalline phases. For example, the transformation of metastable amorphous probucol nanoparticles into crystalline ones was indicated by the gradual increase in the particle stiffness measured by atomic force microscopy (AFM) (Figure 3C) [96,106]. Similarly, the crystallization within transient drug-rich nanodroplets was revealed in the nifedipine-HPMCAS supersaturated solution using NMR (Figure 3D) [107]. Upon the onset of crystallization, the concentration of the drug decreased in the solution, whereas the concentration of polymer increased. Interestingly, these results indicate the HPMCAS polymer was distributed in the drug-rich phase, which might assist the formation of such phase as well as inhibit the crystallization within it.

The transformation of metastable drug-rich phases into crystals is a thermodynamically inevitable process, but the kinetics of this process can be significantly altered by the presence of certain excipients, such that the precipitation of crystalline drugs in ASD solution may not be observed within a prolonged experimental timescale. For example, Keisuke and Lynne suggested that the coalescence of the drug-rich nanodrops could be suppressed by the steric repulsion and electrostatic repulsion after certain polymers are adsorbed on the surfaces [108]. The suppressing effects can be varied by the choice of excipient materials, as demonstrated with the PVP, HPMC and its derivative HPMCAS in the danazol-rich nanodrop system (Figure 3B) [23]. Moreover, many polymers were observed to inhibit the formation of crystalline nuclei or their subsequent growth within the drug-rich phases. Interestingly, Prateek and Ronald investigated the drug and excipient performance in aqueous solutions using all-atom molecular dynamics simulations, where the effects of excipient on the mobility of drugs can be screened [109]. They found that the aggregation and diffusivity of the phenytoin drug can be reduced by as much as five orders of magnitude by the presence of HPMC and HPMCAS, indicating a significant role of excipient in reducing the transformation kinetics of metastable drug-rich phases during the dissolution of ASD.

**Table 2:** Reported drug-rich phases during ASD dissolution over the last decade.

Drugs	Drug Weight Fraction (%)	Excipients	Drug-rich Phase Formation Concentration (μg/mL)	Size (nm)	Metastable Phase ^a^	Apparent Solubility/Binodal Point Concentration	References
Ritonavir	10	PVP	27–28.5	/	1	0.69	[93,95,110]
		PVPVA	26.8–27.5	/	1	2.96	
		HPMCAS	27.5	/	1	1.27	
	50	PVP	27–28.5	/	0	0.69	
		PVPVA	26.8–27.5	/	0	0.37	
		HPMCAS	27.5	/	1	0.76	
	10	pure drug	18.2	188–830	/	∼1	
		PVP	18.3	188–631	1	∼1	
		PAA ^b^	18.3	213–922	1	∼1	
		HPMC	18.7	203–714	1	∼1	
		HPMCAS	18.2	235–352	1	∼1	
		CAAdP 0.85 ^c^	21.3	203–248	1	∼1	
	10–30	PVPVA	∼30	218	1	8.67–3.33	
	35–50			/	0	0.3–0.5	
clotrimazole	/	pure drug	490.2 (pH 4)	100–400	1	0.98	[72]
			7.7 (pH 8)		1	0.87	
nicardipine	/	pure drug	105.9 (pH 6)	100–400	1	1.1	[72]
			5.5 (pH 9)		1	0.69	
atazanavir	/	pure drug	668.4 (pH 3.5)	100–400	1	0.95	[72,111]
			65.8 (pH 9)		1	0.91	
	/	pure drug	/	204–226	/	/	
	10	HPMCAS	78	294	1	3.21	
	30			/	0	1.28	
	50			/	0	1	
	10	HPMCS	78	326	1	2.95	
	30			/	0	1	
	50			/	0	1	
	10	PVPVA	94	/	0	1.17	
	30			/	0	0.96	
	50			/	0	0.74	
danazol	/	pure drug	8.0 (estimated)	267	/	/	[112]
	10	PVP	mean 6.0	256	1	1.58	
	50		mean 8.5	/	1	1.12	
	10	HPMC	mean 8.5	284	1	1.18	
	50		mean 8.5	/	1	1.18	
	10	HPMCAS	mean 6.5	246	1	2.54	
	50		8.0 (estimated)	/	0	0.75	
nilvadipine	5–10	PVPVA	30–31.9	237–246	1	∼3	[113]
	15–20			/	0	∼0.5	
cilnidipine	5–15	PVPVA	0.5–0.6	255–366	1	∼158	[113]
	20–25			/	0	∼1–2	
glibenclamide	33.3	HPMC	∼150	/	0	1	[114]
		HPMCAS-LF	∼150	/	1	2.4	
		HPMCAS-HF	∼150	/	0	1	
enzalutamide	/	pure drug	42–43	/	/	/	[53]
	10	PVPVA	42	/	1	1	
	50			/	0	0.95	
	10	HPMCAS	43	/	/	0.24	
	50			/	/	1	
Lopinavir	50	HPMC	17.4	/	/	1	[70]
itraconazole	25	HPMCAS-HF	∼0.1	/	1	1600	[54,115]
		HPMCAS-LF	∼0.1	/	1	4500	
		HPMCAS 716HP	0.1 (0% SIF^l^)	170	1	4530	
			6 (0.5% SIF^l^)	150	1	81.16	
			20 (2% SIF^l^)	200	1	31.1	
		HPMCAS 126HP	0.1 (0% SIF^l^)	140	1	1540	
			6 (0.5% SIF^l^)	160	1	31	
			20 (2% SIF^l^)	170	1	8.5	
		HPMCAS 716HP; HPMCAS HF	0.1 (0% SIF^l^)	210	1	4030	
			6 (0.5% SIF^l^)	200	1	93.33	
			20 (2% SIF^l^)	190	1	29.05	
Telaprevir	10	PVPVA	100	156	1	1.22	[116]
	30				0	0.8	
	50				0	0.75	
	10	HPMC	96	147	1	1.88	
	30				1	1.1	
	10	HPMCAS	102	99	1	1.57	
	30				1	1.17	
	50				0	0.88	
	50	HPMCAS + 5% SDS ^d^			1	1.08	
	10	CA Sub ^e^	111	76	1	1.44	
phenytoin	10	HPMCAS	/	15	1	/	[105,117]
	25			15	1	/	
	50			/	0	/	
	10	C2-PNIPA-m-7 ^f^	/	1.8–2.0 (pure polymer)	1	<1	
		C12-PNIPA-m-7 ^f^	/	7.6–7.9(pure polymer)	1	<1	
		C12-PNIPA-m-30 ^f^	/	12.0–12.7 (pure polymer)	1	<1	
		C12-PNIPA-m-98 ^f^	/	24.5–32.1;7.9–8.6 (pure polymer)	1	<1	
probucol	10	HPMCAS	<1	16–20	1	/	[96,105,106,118,119,120]
	25			70	1	/	
	50			180	1	/	
	25	HPMC; SDS (weight ratio of 1.75:1.25)	<1	25–93.9 (0–7days)	1	>500	
	14.3	HPMC; SDS (weight ratio of 2:1)	<1	25.3–138.3 (0–12days)	1	>500	
	0	PDMA	/	14.5	1	<1	
	10			54	1	<1	
	25			6.3	1	<1	
	50			13.1	1	<1	
	0	P(DMA-grad-MAG)	/	35	1	<1	
	10			48	1	<1	
	25			61	1	<1	
	50			8	1	<1	
	0	PEP-PDMA	/	25.8	1	<1	
	10			25.1	1	<1	
	25			65	1	<1	
	50			77	1	<1	
	10;25	PND34-C2	∼2000 (without excipient)	5.7	1	<1	
		PND34-C12		15.9	1	<1	
		PND34-b-PS2-C12		19.7	1	<1	
		PND34-b-PS9-C12		22.7	1	<1	
		PND34-b-PS14-C12		27.0	1	<1	
	10	PNIPAm; 5.0 mol % BIS ^h, i^	/	42–46	1	/	
		PNIPAm; 2.5 mol % BIS ^h, i^		43–44	1	/	
		PNIPAm; 0.5 mol % BIS ^h, i^		41–43	1	/	
nilutamide	10;25	PND34-C2	∼1400 (without excipient)	5.7	1	<1	[119]
		PND34-C12		15.9	1	<1	
		PND34-b-PS2-C12 ^g^		19.7	1	<1	
		PND34-b-PS9-C12 ^g^		22.7	1	<1	
		PND34-b-PS14-C12 ^g^		27.0	1	<1	
nifedipine	10	HPMC-E5 LV ^j^	110–156 (5–25 °C, without excipient)	∼200	1	1.05	[121]
	20				1	1.15	
	10	PVPVA			1	1.1	
	20				1	1	
anacetrapib	20	copovidone; TPGS	<1	50–200 (10–2% TPGS) ^k^	1	∼90	[122,123]
						/	

^a^ 1 represents the presence of the metastable phase, 0 represents the absence of the metastable phase; ^b^ PAA represents the poly(acrylic acid); ^c^ CAAdP 0.85 represents the cellulose acetate propionate 504–0.2 adipate 0.85; ^d^ SDS represents the Sodium dodecyl sulfate; ^e^ CA Sub represents the cellulose derivative cellulose acetate suberate; ^f^ C12-PNIPA-m represents the dodecyl (C12)-tailed poly(Nisopropylacrylamide); ^g^ PND-b-PS-C12 represents the poly(N-isopropylacrylamide-co-N,N-dimethyl- acrylamide)-b-polystyrene; ^h^ BIS represents the N,N′- methylenebis(acrylamide); ^i^ PNIPAm represents the poly(N-isopropylacrylamide); ^j^ HPMC-E5 LV represents the Hydroxypropyl methylcellulose E5 Premium LV; ^k^ TPGS represents the D-α-tocopheryl polyethylene glycol 1000 succinate; ^l^ SIF represents the bile salt.

## 3. In Vitro Permeability Enhancement Achieved by the ASD Solution

### 3.1. Drug Solubility-Permeability Interplay

**Figure 4:**
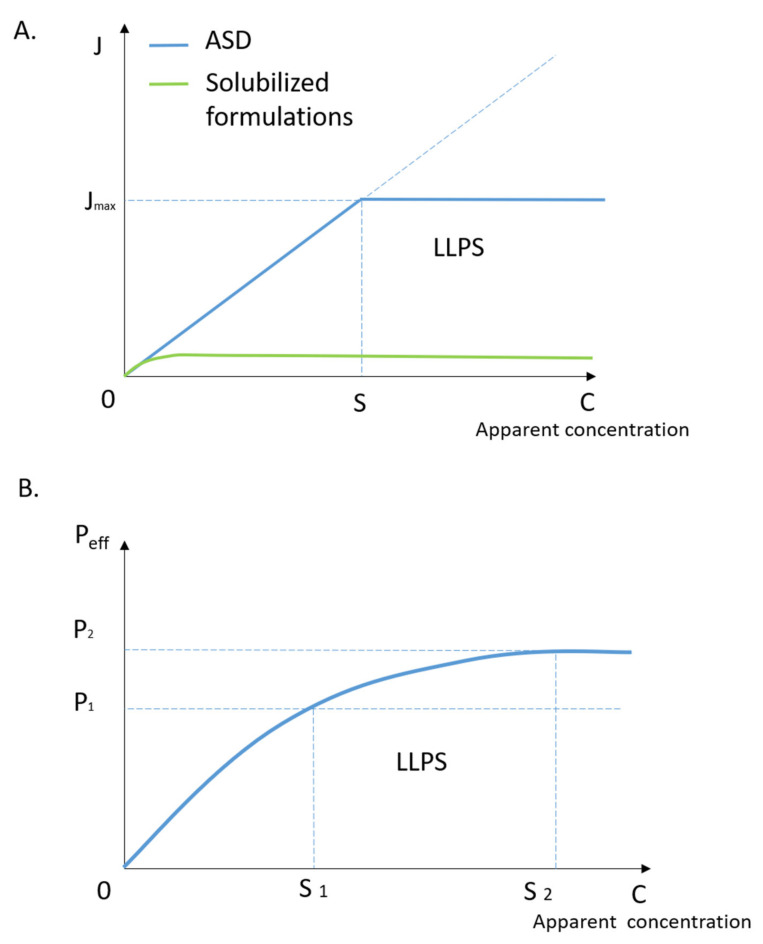
(**A**) Schematic diagram of the drug flux across the membrane as a function of apparent concentration. The blue line represents the theoretical drug flux across the membrane for ASD and the green line represents that of solubilizing formulations, S represents the concentration where the drug-rich phase forms. (**B**) The schematic diagram of permeability with the increasing of apparent concentration in the donor compartment with considering the effect of the drug-rich phase on the effective thickness of ABL. S1 represents the concentration at which the drug-rich phase forms, S2 represents the maximum effective concentration for the drug-rich phase in respect to the permeability enhancement.

In the past decades, various enabling formulations were developed to enhance the delivery of poorly water-soluble drugs; a reduction instead of an enhancement of the oral absorption was often observed for certain formulation strategies [124,125,126,127]. The lack of correlation between solubility and permeability is known as the “solubility-permeability interplay”, resulting in the increased apparent solubility alone not being sufficient to predict and evaluate the drug’s oral absorption [128,129,130,131,132]. The true driving force of mass transport through the membrane has been widely investigated as an efficient tool for the development of poorly water-soluble drugs. Importantly, the drug’s thermodynamic activity was suggested to be the driving force of the increased membrane permeability [133]. The relationship between the drug influx across the membrane and its thermodynamic activity has been described by Raina et al. [133]:

(1) $$J=\frac{dM}{dt}=\frac{{DAa}_{aq}}{\gamma_{m}h}$$

where J represents the drug flux across the membrane, dM/dt is the change of solute mass in unit time. The drug flux depends on the drug diffusion coefficient (*D*), membrane area (*A*), drug thermodynamic activity in the aqueous solution (*a_aq_*), activity coefficient of the drug in the membrane ($\gamma_{m}$), and the thickness of the membrane (*h*). If we define:

(2) $$B=\frac{DA}{\gamma_{m}h}$$

with the hypothesis that excipients in ASD will not alter the parameters of *D*, *A*, $\gamma_{m}$ and *h*, the changing of the drug flux is proportional to the drug’s thermodynamic activity:

(3) $$J=B\cdot a_{aq}$$

where the *B* is a constant number. In the ASD solution, theoretically, the thermodynamic activity of the drug is associated with the apparent concentration and the activity coefficient of the drug in the aqueous solution. Thus, the thermodynamic activity of the drug increases proportionally to the drug concentration until the binodal point, which indicates the onset of the drug-rich phases (Equation (5)). Therefore, the drug’s thermodynamic activity is obtained as:

(4) $$\left\{\begin{array}{c} \;C<S\\ a_{aq}=\gamma_{aq}\cdot C \end{array}\right.$$

(5) $$\left\{\begin{array}{c} \;C⩾S\\ a_{aq}=\gamma_{aq}\cdot S \end{array}\right.$$

where *C* is the drug’s apparent concentration and *S* is the critical apparent solubility of the drug corresponding to the onset of LLPS, $\gamma_{aq}$ is the activity coefficient of the drug in the aqueous donor solution. According to Equations (3)–(5), the drug influx across the membrane as a function of the drug’s apparent concentration in the system may be illustrated by the blue line in Figure 4A. The flux initially increases with the increase in the drug’s apparent concentration and reaches a plateau at a critical drug concentration where the drug-rich phase forms, indicating the maximum permeability in correlation to the critical apparent solubility of the drug in ASD. Based on this relation, other solubilization techniques by forming drug-containing micelles or drug-excipient complexes were introduced to further increase the critical apparent solubility of the drug in the ASD system [134]. Surprisingly, a much lower drug influx was observed, as schematically illustrated in the green line in Figure 4A. Indeed, the apparent solubility of the drug is not the only factor that determines the drug influx according to Equations (3)–(5). Other factors, such as the drug diffusion coefficient (*D*), activity coefficient in the membrane ($\gamma_{m}$) or in the donor solution ($\gamma_{aq}$), membrane area (*A*) or thickness (*h*) can also play important roles. For example, the solubilization technique of using surfactant might enhance the apparent solubility of the drug by significantly sacrificing its activity coefficient in ASD and during the membrane absorption. By contrast, the ASD formulation typically enhances the apparent solubility of the drug via the formation of the drug-rich metastable phase that does not compromise the activity coefficient of the drug. This contrasting effect is a likely explanation for the striking differences demonstrated in Figure 4A. However, more experimental evidence is still required to validate such an explanation.

### 3.2. The Roles of Drug-rich Phase in Membrane Transportation

The overall enhancement of drug permeability and bioavailability has been frequently reported with the presence of a drug-rich metastable phase during the dissolution of ASD formulations [56,123,135,136]. This metastable phase can temporarily enhance the drug’s apparent solubility and, therefore, the drug influx [127,130]. Apart from the enhanced apparently solubility, drug-rich phases are suggested to further increase oral bioavailability via two other possible mechanisms. During the drug’s oral absorption, with the free drug molecules diffusing across the epithelial membrane, the drug from the metastable phase can easily re-dissolve and maintain the overall drug concentration within the GI fluid. This is known as the “replenish” or “reservoir” mechanism [52,53]. Furthermore, drug nanoparticles or nanodrops could reduce the effective thickness of the aqueous boundary layer (ABL) next to the surface of the intestinal membrane and increase the drug concentration as a “shuttle” or “drifting” mechanism [54,55,56,57]. The ABL is an obstacle for permeation of the drugs, especially for the hydrophobic drugs for which the diffusivity across the ABL is lower than absorption through the membrane. The diffusivity of the ABL can be increased by enriching the drug’s apparent concentration through the formation of the drug-rich phase [54,115,122,137,138]. These species can ultimately enhance the permeability (*P_ABL_*) of the drug through the ABL and lead to the enhancement of the overall effective permeability (*P_eff_*). The overall effective permeability (*P_eff_*) is defined by both drug permeability through the membrane (*P_m_*) and the apparent aqueous boundary layer (*P_ABL_*) described as:

(6) $$P_{eff}=\frac{1}{\frac{1}{P_{m}}+\frac{1}{P_{ABL}}}$$

**Figure 5:**
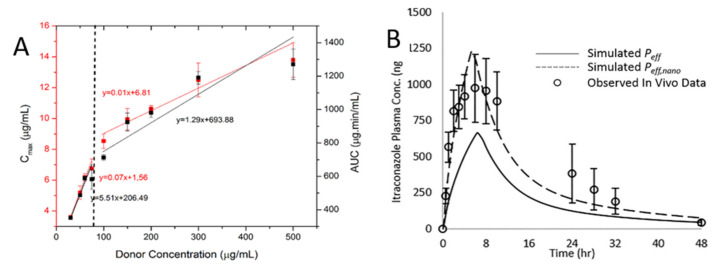
(**A**) The maximum Atazanavir (ATZ) concentration (Cmax) and the area under the curve (AUC) values plotted with respect to the initial donor concentration. The vertical line represents the drug’s amorphous solubility. Reproduced with permission from [111], American Chemical Society, 2018. (**B**) A comparison of the itraconazole in vivo data and permeability models with or without modified with the effect of nanoparticles in aqueous solution “drifting” into the ABL. Reproduced with permission from [139], American Chemical Society, 2019.

Considering that the effect of the drug-rich phase enhances the drug permeability through the ABL, the schematic diagram of drug flux versus drug apparent concentration may be illustrated in Figure 4B. S_1_ represents the concentration at the occurrence of the drug-rich phase and S_2_ is the maximum effective concentration for the drug-rich phase in respect to the permeability enhancement. P_1_ and P_2_ are drug permeability at corresponding concentrations, respectively. The driving force of the increasing permeability from 0 to P_1_ is achieved by the drug’s apparent solubility. The slop is altered with the concentration increasing from S_1_ to S_2_ due to the change of permeability mechanism. The dominant factor for effective permeability (*P_eff_*) enhancement is shifted from P_m_ to *P_ABL_* as the apparent drug concentration reaches S_1_ and beyond. Theoretically, with the presence of the drug-rich metastable phase and appropriate nanoparticle/nanodrop size, drug permeability will increase until the unbound amorphous drug is saturated at the surface of the membrane. The maximum permeability then reaches a new plateau at a high apparent drug concentration. Experimentally, Siddhi et al. demonstrated the dissolution performance and membrane mass transportation for atazanavir (ATZ) ASD using a high surface area apparatus [111]. Figure 5A illustrated the maximum drug concentration (C_max_) and the area under the curve (AUC) values in the acceptor compartment in relation to the drug’s apparent concentration in the donor compartment. A clear step-change on the drug membrane influx can be observed at the concentration value of the ATZ-rich metastable phase, indicating the potential benefits of such metastable phases during the dissolution of ASD. Aaron and Michael further evaluated this drug-rich phase-induced transport enhancement mechanism with a modified permeability model [139]. Accounting for the effects of the drug-rich phase on diffusivity across the ABL, the experimental behaviors of itraconazole ASD absorption in vivo was successfully described, as illustrated in Figure 5B. Similarly, a novel approach was developed by Freddy et al. for accurately measuring the thermodynamic activity of drugs and predicting their true flux with the presence of drug-rich nanodroplets [140].

### 3.3. The Importance of Polymeric Excipients for Drug Permeability Enhancement

It is worth highlighting that the drug permeability enhancement for ASD formulation heavily relies upon the properties of polymeric excipients. Through rational selection of polymers, additional benefits, in terms of the drug’s oral bioavailability, could be achieved through the formation of the drug-rich metastable phase; however, a plateau may be reached when an excess amount was used. For example, in relation to the Eudragit^®^ E, the equilibrium solubilities of bezfibrate, indomethacin, piroxicam, and warfarin were increased proportionally with the increases in the polymer concentration up to 2% (*w*/*w*) until a plateau is reached [141,142]. Besides, when a strong interaction is formed between drug and polymeric excipients, such as ionic interaction, the solubility-permeability tradeoff occurs [143,144]. Similarly, the drug-polymer molecular complex can negatively impact the drug’s membrane permeability. In the phenytoin-PVP system at polymer concentrations above 0.5 mg/mL, a strong interaction was observed, presenting a downfield shift of the phenytoin proton peak (NMR) [145]. Such interaction between drug and polymer can lead to the electron-withdrawing effect, resulting in a decrease in apparent permeability coefficients for corresponding drugs. In contrast, polymers stabilizing the supersaturated drug solution without significantly affecting the thermodynamic activity may result in a better permeability enhancement.

Processing ASD into nanoparticles as means of formulation has also been frequently considered as a desirable strategy for increasing the therapeutical windows of highly potent compounds [61,146,147,148,149,150]. Examples of amorphous drug-polymer nanoparticle formulations exhibiting enhanced in vitro and in vivo performances are summarized in Table 3. Formulations were prepared using techniques such as antisolvent, ionic cross-linking, solvent displacement, spray drying, freeze-drying, and twin-screw extrusion, etc., with mean sizes ranging from 50 to 500 nm. Small fractions of surfactants were additionally added to further reduce the particle sizes and improve stability. Such a formulation strategy may be considered for similar species in the drug-rich phase generated by ASD, with added benefits such as targeted or site-specific drug release as well as penetration of the mucus layer within the body [151]. For example, the amorphous drug-polymer system is capable of controlled release of small molecules and large biological molecules to mucosal surfaces, such as those of the lung airways, GI tract, female reproductive tract, nose, and eye; therefore, it is of widespread interest in the field of nanomedicine [146,151,152,153]. Muco-penetration particles are aimed to slip through the mucus with the modification of the particle surface, especially surface charge and viscosity [147]. Due to the overall negative charge of the mucus, formulations with noninteracting polymers, high-charge density net-neutral particles, charge-shifting particles are designed for better penetration of the mucus layer [148,149,154]. On the other hand, the mucoadhesive drug delivery system (MDDS) featuring a longer adhesion time and subsequent penetration through the epithelial membrane is also a desirable formulation design. Polymers such as HPMC, thiolated polymers, polysaccharides, or maleimide modified polymers were reported for the MDDS [61,150,155,156,157].

## 4. Conclusions

The supersaturated ASD solution increases the oral bioavailability of drugs through the elevation of both the drug’s apparent solubility and its permeability. We suggest that such enhancement is likely to be contributed by the excipient-assisted formation and stabilization of drug-rich metastable phases following the non-classical nucleation pathway. In this case, a high apparent solubility of a drug could be kept for an extended amount of time in the solution. Therefore, suitable thermodynamic and kinetic models featuring drug-excipient-water ternary phase behaviors may be useful for designing the ASD formulation with the ability of self-generating and kinetically stabilizing drug-rich phases. Furthermore, these drug-rich amorphous phases, might significantly increase the drug’s oral absorption by other mechanisms such as (i) the “reservoir” mechanism, in which the drug is replenished when the drug concentration in the GI tract or the donor compartment is lower than its solubility; (ii) the increasing of the drug concentration on the surface of the membrane by a “shuttle” effect; (iii) potentially sliding into the mucus layer that is adjacent to the intestinal membrane. Designs of ASD formulations that enable the synergetic effects of these different mechanisms to enhance the drug oral absorption will be highly beneficial for future medicine development.

## Figures and Tables

**Table 1:** Summary of ASD products granted FDA approval from 2015 to 2020.

Brand Name	Generic Name	Company	Manufacture Technique	FDA Approval	Dosage Form
Kalydeco^®^	ivacaftor	Vertex	SD	2015	granule
Orkambi^®^	lumacaftor; ivacaftor	Vertex	SD	2015	tablet
Epclusa^®^	sofosbuvir; velpatasvir	Gilead Sciences	SD	2016	tablet
Venclexta^®^	venetoclax	AbbVie	HME	2016	tablet
Viekira XR™	dasabuvir sodium; ombitasvir; paritaprevir; ritonavir	AbbVie	HME	2016	tablet
Zepatier^®^	elbasvir; grazoprevir	Merck	SD	2016	tablet
Lynparza^®^	olaparib	Astrazeneza	HME	2017	tablet
Norvir^®^	ritonavir	AbbVie	HME	2017	powder
Mavyret™	glecaprevir; pibrentasvir	AbbVie	HME	2017	tablet
Prograf^®^	tacrolimus	AbbVie	SD	2018	granule; capsule
Tibsovo^®^	ivosedinib	AbbVie	/	2018	tablet
symdeko^®^	tezacaftor; ivacaftor	Vertex	/	2018	tablet
Trikafta^®^	elexacaftor; tezacaftor; ivacaftor	Vertex	/	2019	tablet
Harvoni^®^	ledipasvir; sofosbuvir	Gilead Sciences	SD	2019	pellet

This summary is adapted from several references [26,27,28,29]. Details of the approval year, active ingredients, companies and dosage forms are derived from the FDA drug database and annual approval reports [17]. SD represents spray drying, HME represents hot melt extrusion.

**Table 3:** Examples of amorphous drug-polymer nanoparticle formulations reported to enhance in vivo drug absorption (from 2010).

Drugs	Excipients	Particle Size	Comments	References
heparin	thiolated chitosan; HPMCP	200–500nm (1360 nm for the special drug-excipient ratio)	A significant improvement of mucoadhesion was observed in rats; a 1.86-fold improvement of permeation-enhancing effect was observed in freshly excised carp intestine.	[156]
celecoxib	PVP K30; TPGS	less than 300 nm	The amorphous nanoparticles showed 4.6- and 5.7-times greater AUC _0–24 h_ and C_max_ in plasma, respectively, compared with the unprocessed form.	[158]
celecoxib	HPMC E5 and SDS (2:1, w/w)	mean 159 nm	The maximum drug concentration and AUC_0–24h_ of amorphous celecoxib nanoparticles were observed to be 3-fold and 2-fold greater than those of the celecoxib capsules.	[159]
silibinin	HPMC	132.3 nm	The dissolution rate of silibinin nanoparticles was 48.2 times and 153.8 times higher than that of free silibinin in gastric juice and intestinal juice, respectively. The C _max_ and AUC values for silibinin nanoparticles were 15.3 times and 6.48 times greater than those in the free drug.	[160]
bezafibrate	PVP K30; cremophor ELP	less than 500 nm	Smooth-surfaced amorphous particles exhibited a 5.5-fold higher oral bioavailability compared with the plain bezafibrate powder.	[161]
valsartan	HPMC; poloxamer 407	less than 400 nm	Valsartan-HPMC-surfactant nanoparticles increased the drug release speed (up to 90% within 10 min), exhibited 7.2-fold greater maximum plasma concentration, and 4.6-fold higher AUC_0–24h_.	[162]
cyclosporine A	HPMCP; including HP50 and HP55	50–60 nm	Significant sustain drug release; the bioavailability of pH-sensitive drug nanoparticles calculated by the AUC_0–72h_ was 119.6% for HPMC HP55.	[163]
raloxifene	PVP	180 nm	The raloxifene loaded suspension nanoparticles were found to be 3.3-fold and 2.3-fold higher, in terms of AUC and C_max_ increment, respectively, in plasma than those of the drug powder.	[164]
anacetrapib	copovidone; vitamin E TPGS	/	Particles with sizes of less than 100 nm were observed to have approximately 2-fold higher average exposure in dogs compared with larger particles.	[123]
7-ethyl10-hydroxycamptothecin (SN-38)	disodium glycyrrhizin	69.41 nm	The amorphous formulation increased the drug solubility of the crystal drug 189-fold. The nanoparticles exhibited 4-fold greater bioavailability than that of drug suspension.	[165]
tadalafil	HPMC, VA64, and PVP K30	200 nm	The amorphous particles exhibit 8.5 times faster dissolution rates in the first minute of dissolution, 22-fold greater apparent solubility at 10 min, 3.67-fold greater in oral bioavailability than unprocessed tadalafil.	[166]
megestrol acetate	HPMC; PVPK30; Ryoto sugar ester L1695	less than 500 nm	The solid dispersion nanoparticles showed 4-fold and 5.5-fold higher AUC_0–24h_ and C_max_ than those of raw drug powder.	[167]
sitagliptin	chitosan	210–618 nm	The chitosan-loaded amorphous nanoparticles showed a sustained release and mucoadhesive properties.	[157]
itraconazole	HPMCAS 716HP; HPMCAS 126HP	140–210 nm	The drug-rich nanoparticles have been demonstrated contribution to the ABL diffusion in proportion to their diffusion coefficients and drug loadings.	[115]
sirolimus	PVP K30; surfactants	mean 250 nm	The peak concentration and AUC_0-12h_ of sirolimus were increased 18.3-fold and 15.2-fold, respectively.	[168]
enzalutamide	HPMCAS; PVPVA	42–43 nm	ASDs that formed drug nanoparticles during dissolution showed higher drug concentration for rat plasma exposure than samples that only yielded supersaturated solutions.	[53]

* HPMCP represents hydroxypropyl methylcellulose phthalate; TPGS represents d-α-tocopheryl polyethylene glycol 1000 succinate; Cmax represents the maximum drug concentration; AUC 0-t represents the area under the concentration-time curve over time (t).

## Data Availability

Not applicable.
